# Human‐specific polymorphic pseudogenization of *SIGLEC12* protects against advanced cancer progression

**DOI:** 10.1096/fba.2020-00092

**Published:** 2020-12-08

**Authors:** Shoib S Siddiqui, Michael Vaill, Raymond Do, Naazneen Khan, Andrea L Verhagen, Wu Zhang, Heinz‐Josef Lenz, Teresa L Johnson‐Pais, Robin J Leach, Gary Fraser, Charles Wang, Gen‐Sheng Feng, Nissi Varki, Ajit Varki

**Affiliations:** ^1^ Departments of Medicine, Cellular and Molecular Medicine, and Pathology, Glycobiology Research and Training Cente and Center for Academic Research and Training in Anthropogeny University of California San Diego CA USA; ^2^ University of Southern California Norris Comprehensive Cancer Center Los Angeles CA USA; ^3^ Department of Urology University of TX Health Science Center San Antonio TX USA; ^4^ Departments of Cell Systems and Anatomy University of TX Health Science Center San Antonio TX USA; ^5^ School of Public Health Loma Linda University Loma Linda CA USA; ^6^ Present address: Department of Biotechnology American University of Ras Al Khaimah (AURAK American University of Ras Al Khaimah Road Al Burairat Area Ras Al Khaimah UAE

**Keywords:** advanced carcinoma, dot blot, immunohistochemistry, pseudogenization, *SIGLEC12*

## Abstract

Compared with our closest living evolutionary cousins, humans appear unusually prone to develop carcinomas (cancers arising from epithelia). The *SIGLEC12* gene, which encodes the Siglec‐XII protein expressed on epithelial cells, has several uniquely human features: a fixed homozygous missense mutation inactivating its natural ligand recognition property; a polymorphic frameshift mutation eliminating full‐length protein expression in ~60%–70% of worldwide human populations; and, genomic features suggesting a negative selective sweep favoring the pseudogene state. Despite the loss of canonical sialic acid binding, Siglec‐XII still recruits Shp2 and accelerates tumor growth in a mouse model. We hypothesized that dysfunctional Siglec‐XII facilitates human carcinoma progression, correlating with known tumorigenic signatures of Shp2‐dependent cancers. Immunohistochemistry was used to detect Siglec‐XII expression on tissue microarrays. PC‐3 prostate cancer cells were transfected with Siglec‐XII and transcription of genes enriched with Siglec‐XII was determined. Genomic *SIGLEC12* status was determined for four different cancer cohorts. Finally, a dot blot analysis of human urinary epithelial cells was established to determine the Siglec‐XII expressors versus non‐expressors. Forced expression in a *SIGLEC12* null carcinoma cell line enriched transcription of genes associated with cancer progression. While Siglec‐XII was detected as expected in ~30%–40% of normal epithelia, ~80% of advanced carcinomas showed strong expression. Notably, >80% of late‐stage colorectal cancers had a functional *SIGLEC12* allele, correlating with overall increased mortality. Thus, advanced carcinomas are much more likely to occur in individuals whose genomes have an intact *SIGLEC12* gene, likely because the encoded Siglec‐XII protein recruits Shp2‐related oncogenic pathways. The finding has prognostic, diagnostic, and therapeutic implications.

**Abbreviations**
BCR: Biochemical recurrence
BST2: Bone Marrow Stromal Cell Antigen 2
CEACAM6: Carcinoembryonic Antigen‐Related Cell Adhesion Molecule 6
CTSF: Cathepsin F
CXADR: Coxsackievirus and adenovirus receptor
GSEA: Gene set enrichment analysis
IDO1: Indoleamine 2,3‐Dioxygenase 1
ITIM: Immunoreceptor tyrosine‐based inhibitory motifs
LCP1: Lymphocyte Cytosolic Protein 1
MSigDB: Molecular Signature Database
NED: No evidence of disease
PRLS: post‐reproductive life span
PSA: Prostate‐specific antigen
Siglec: Sialic‐acid‐binding immunoglobulin‐like lectins
TACSTD2: Tumor‐Associated Calcium Signal Transducer 2
ZNF43: Zinc Finger Protein 43

## 1 INTRODUCTION

Humans appear unusually prone to develop carcinomas (cancers arising from epithelial cells), compared with our closest evolutionary cousins (“great apes”).^1^, ^2^, ^3^ Here, we show an unexpected human‐specific connection between advanced carcinomas and a member of the CD33‐related family of Siglecs (Sialic acid‐binding Ig‐like lectins) receptors.^4^, ^5^, ^6^ Siglecs are cell‐surface receptors typically expressed on innate immune cells, and binding ligands bearing sialic acids (Sias), a family of glycans prominently present at the terminal end of the glycan chains on cell surface and extracellular glycoconjugates.^7^ Most CD33‐related Siglecs (CD33rSiglecs) have immunoinhibitory functions mediated by immunoreceptor tyrosine‐based inhibitory motifs (ITIMs) and ITIM‐like motifs in the cytosolic tail.^4^, ^5^, ^6^ Upon binding sialic acid ligands, these intracellular signaling motifs recruit protein tyrosine phosphatases Shp1 and Shp2, which subsequently participate in a variety of signaling pathways in immune cells to influence cellular activation.^4^, ^5^, ^6^

Unlike other CD33‐related Siglecs, the literature on Human Siglec‐XII (encoded by the gene *SIGLEC12*) is sparse.^8^, ^9^, ^10^, ^11^, ^12^ In fact, it has been largely ignored and even excluded from major reviews on Siglec biology,^6^, ^13^, ^14^, ^15^ because the protein and the locus encoding *SIGLEC12* are atypical in several ways. First, the protein has two amino‐terminal V‐set domains,^9^ compared with only one in all other Siglecs. Second, there is a human‐universal mutation of critical arginine residues in both V‐set domains, rendering it unable to recognize Sias (hence, the use of the Roman numeral XII for the protein, instead of Arabic numerals for functional Siglecs). Third, the Arg––>Cys mutation of the V‐set 1 domain is not present in orthologs of closely related “great apes” (chimpanzee, baboon, gorilla, and orangutan).^8^ Fourth, chimpanzee Siglec‐12 preferentially recognizes a form of Sia (Neu5Gc) that was lost from the human lineage due to an independent fixed mutation of *CMAH* ^8^. Fifth, the *SIGLEC12* gene harbors a common polymorphic frameshift mutation causing truncation of Siglec‐XII and/or alternate splicing^16^ that causes loss of expression of full‐length protein^11^ in the majority of humans. Finally, while the wild‐type protein is expressed in some tissue macrophages, it is not found on other blood cell types, and is instead more prominent on epithelial cell surfaces.^11^

At first glance, the above features suggest a nonfunctional protein in the process of being eliminated from humans by pseudogenization. However, forced expression of human Siglec‐XII in a genetically null human carcinoma cell line led to enhanced tumor growth in nude mice.^11^ Furthermore, while human Siglec‐XII does not recognize Sias, it still has ITIM and ITIM‐like domains in the cytosolic tail that can be phosphorylated to recruit Shp1 and Shp2 phosphatases.^9^ Finally, genome‐wide analysis of signals of selection in human populations identified polymorphisms that introduce nonsense‐mediated decay into human genes, including *SIGLEC12*.^17^ The human *SIGLEC12* locus appears to be undergoing selection favoring a null and/or truncated form, a possible example of the “less‐is‐more” hypothesis first proposed by Olson.^18^

Here, we focus on Siglec‐XII expression in tumor and normal epithelia, identify genes upregulated upon Siglec‐XII expression, address the predictive value of *SIGLEC12* status in cancer cohorts, and provide further evidence suggesting ongoing selection for the null state. Finally, we report a simple urine test to screen for the minority of individuals capable of full‐length Siglec‐XII expression.

## 2 MATERIALS AND METHODS

### 2.1 Immunohistochemistry studies

Multi‐tissue array slides were obtained from US Biomax (Rockville, Maryland), which were completely anonymized and consisted of normal human and cancer tissues. A second set of multi‐tissue array slides were obtained from Novus Bio, which contained a variety of malignancies (about 476 different types) and also a set of normal multi‐tissue array. The sections were de‐paraffinized and blocked for endogenous biotin and peroxidase. The heat‐induced epitope retrieval was performed with citrate buffer pH 6. A five‐step signal amplification method was used which includes application of mouse monoclonal anti‐Siglec‐XII antibody (clone 276), followed by biotinylated donkey anti‐mouse, horseradish peroxidase (HRP), Streptavidin, followed by application of the enzyme biotinyl tyramide, and then, labeled Streptavidin. The AEC kit (Vector) was used as substrate, nuclear counterstain was with Mayer's hematoxylin, and the slides were aqueous mounted for digital photographs, taken using the Olympus BH2 microscope.

### 2.2 Buccal swab

Healthy volunteers were recruited, and their buccal swab samples were used for DNA isolation with institutional review board (IRB) approval issued by the University of California, San Diego (UCSD). Before collection of the swab, the donors were asked to remove the mucous layer of their cheek by rubbing sterile gauze against it. Subsequently a sterile cotton tip was rubbed on the inner cheek cells for genomic DNA isolation. Genomic DNA was isolated using the ChargeSwitch Buccal Cell gDNA isolation kit (Invitrogen, Cat No.‐CS11021) according to the manufacturer's instructions. The PCR amplification for *SIGLEC12* gene was performed using the primers: Forward 5′‐CAATGCAGAAGTCCGTGACGGTGCAGG‐3′ and reverse 5′‐AGGATCAGGAGGGGCATCCAAGGTGC‐3′. The Phusion High‐Fidelity Polymerase kit was used according to the manufacturer's instructions. The DNA amplicon was purified using QIAquick PCR purification kit (Qiagen, Cat no.‐28106) and it was sent for sequencing at Eton Bio, San Diego using the sequencing primer: 5′‐CTCTCTCTGGTGTCTCTGATGC‐3′ (reverse).

### 2.3 Dot blot using urine from healthy donors

Healthy volunteers donated 50 ml of first morning urine according to the IRB approved study. The urine sample was centrifuged at room temperature for 10 min at 500xg. The supernatant was removed, and cell pellet resuspended in 100 µl PBS. The sample was applied onto nitrocellulose membrane and immobilized by applying negative pressure. The membrane was blocked using 50% Licor solution (cat no‐927‐40000) +50% PBST (PBS+0.01%Tween). After blocking, primary anti‐Siglec‐XII antibody (clone 1130) was applied at a dilution of 1:100–1:500. This clone of antibody have been used and characterized before.^11^ The primary antibody dilution was performed in 90% Licor Solution +10%PBST and incubation was carried out for 1 hour at RT. The membrane was then washed with 10 ml of PBST three times for 5 min each. After washing, the membrane was incubated with anti‐mouse‐Licor‐800 antibody at a dilution of 1:10000 in 90% Licor Solution +10%PBST. The secondary antibody incubation was performed for 1 hour at RT in dark. After incubation the membrane was washed with PBST three times for 5 min followed by two times with PBS for 5 min. The band on the membrane was visualized by using Licor fluorescence scanning machine. Here, only PBS was used as a negative control and Siglec‐XII‐Fc was used as a positive control.

### 2.4 *SIGLEC12* frameshift mutation in seventh‐day adventist group

The Seventh‐day Adventist group is a diverse population group where the key carcinogenesis risk factors are less prevalent, such as consumption of red meat, alcohol, and smoking. The genomic DNA was isolated from the peripheral blood cells of 53 cancer patients and 54 age‐matched control subjects. The frameshift deletion mutation of *SIGLEC12* was analyzed by first PCR amplifying the *SIGLEC12* locus using the primers 5′‐ACCCCTGCTCTGTGGGAGAGT‐3′ (forward) and 5′AGGATCAGGAGGGGCATCCAAGGTGC‐3′ (reverse). The PCR was performed using Phusion High‐Fidelity Polymerase kit. The amplified product was purified using the QIAquick PCR purification kit (Qiagen, cat no.‐28106) and sent for sequencing to EtonBio, San Diego, USA. The sequencing was performed using the primer: 5′‐CTCTCTCTGGTGTCTCTGATGC‐3′ (reverse).

### 2.5 RNA‐sequence analysis

PC‐3 and PC‐3‐SigXII expressing cells were cultured to confluency in T25 flasks and mRNA was extracted from the cells using the Qiagen RNeasy plus mini kit (Cat no.‐ 74134). Transcriptomic analysis was performed on RNA libraries prepared from *SIGLEC12* and control PC3 cells using the TruSeq RNA Library Prep Kit v2. Each cell line was used to prepare four separate technical replicate libraries for sequencing. Libraries were sequenced at 1 × 50 bp on HiSeq 4000 (Illumina). Reads were mapped to human reference genome Hg19 using STAR v2.5.3a.^19^ Mapped reads were counted at the gene level using featureCounts v1.5.2^20^ and counts were analyzed using DESeq2 v1.14.1.^21^ Differentially expressed genes with a *p* value ≤0.05 and fold change ≥2 were then selected for further examination and gene set enrichment analysis using the GSEA software,^22^ the MSigDB v7.0 oncogenic signatures collection (C6),^23^ and the Siglec‐XII or Shp2 expression status as phenotype with 1,000 permutations.

### 2.6 Statistical analysis

Graph Prism pad 5.0 was used. The chi‐square test was performed on immunohistochemistry data, different cancer cohorts and a *p* value <0.05 was considered as significant. For the RNA‐Seq the two‐way ANOVA was used as the statistically significant value. The *p* value <0.05 and fold change of 2 was used as a cutoff for assessing the differentially expressed genes.

### 2.7 Population genetics analysis

Human genomes were accessed from the 1000 Genomes Project server (www.1000genomes.org/). Bed coordinates defining the *SIGLEC12* genomic regions were retrieved from build hg19 using the University of California, Santa Cruz (UCSC), genome browser. A region containing *SIGLEC12* gene in three different populations of West Africa, Northern European, and East Asian ancestry (YRI, CHB, and CEU), using the selection tools pipeline.^24^ Statistical tests such as frequency‐based method (Tajima's *D*) and population differentiation‐based methods (F*_ST_*) among three different populations were analyzed.^24^ Each test is suited to detect selection at different timescales. Tajima's *D* is a commonly used summary of the site‐frequency spectrum (SFS) of nucleotide polymorphism data and is based on the difference between two estimators of θ (the population mutation rate 4*Ne*µ): nucleotide diversity that is the average number of pairwise differences between sequences, and Watterson's estimator, based on the number of segregating sites. A negative Tajima's D signifies an excess of low‐frequency polymorphisms, and indicates a population size expansion, selective sweep, and/or positive selection, or negative selection. A positive Tajima's D value indicates a decrease in population size and/or that balancing selection.^25^ On the contrary, the estimator of population differentiation (F*_ST_*), compares the variance of allele frequencies within and between populations.^26^ While large values of F*_ST_* at a locus indicate complete differentiation between populations, which suggests directional selection, small values indicate the lack of differentiation in populations being compared, which might be an indicator of directional or balancing selection in both.^27^ Human genome raw data for *SIGLEC12* ^28^ was utilized for detecting Selective Sweep using SweepFinder2^29^ which implements a composite likelihood ratio (CLR) test.^30^ The CLR uses the variation of the SFS of a region to compute the ratio of the likelihood of a selective sweep at a given position to the likelihood of a null model without a selective sweep. Tajima's *D* and sweep scans were visualized in Excel and F*_ST_* were visualized in R studio platform and examined for evidence of deviation from the null expectation.

## 3 RESULTS

### 3.1 Expression of genes associated with cancer progression in a Siglec‐XII expressing prostate cancer cell line

**Figure 1:**
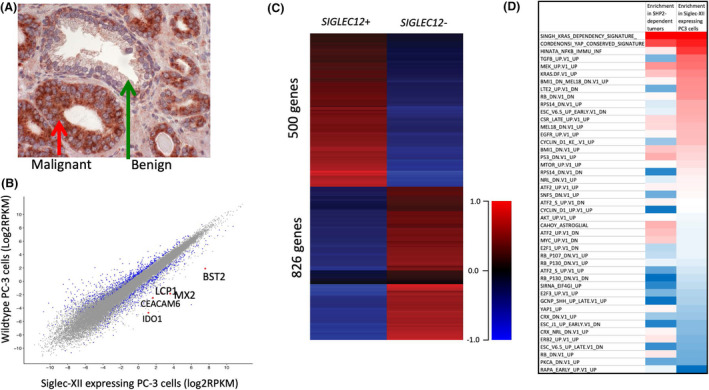
Siglec‐XII Induction of expression of genes associated with cancer progression. A, Example of tissue sections with adjacent normal and malignant cells from a prostate cancer patient. B, Gene Expression in Siglec‐XII transfected prostate cancer cells versus sham transfection (n = 4). Differentially expressed genes highlighted in blue, and genes not differentially expressed are in grey color. A fold change of 2 and *p* value <0.05 was used as a cutoff. C, The heatmap shows the differentially expressed genes in the Siglec‐XII expressing PC‐3 cell line versus parental PC‐3 cells (n = 4). D, Siglec‐XII GSEA shows same top pathway expression as Shp2 positive tumors

Supporting the relevance of Siglec‐XII expression in advanced cancer, we noted that in tissue sections where both malignant and adjacent normal tissue were present, Siglec‐XII expression was higher in the malignant cells (one such example is shown in Figure 1A). To begin to explore the mechanism of action of this cell‐surface receptor, we took advantage of our earlier model system, Siglec‐XII nonexpressing PC‐3 prostate carcinoma cells, which were transfected with a vector causing expression of full‐length Siglec‐XII. We had already observed larger tumors when this PC‐3‐Siglec‐XII cell line was injected subcutaneously into the flanks of athymic nude mice, as compared to PC‐3 cells transfected with vector alone.^11^ We now compared the RNA expression profiles between these two cell lines and found many genes to be differentially expressed (Figure 1B,C). Importantly, these differentially expressed genes were enriched for those known to play a role in cancer biology. A few of those upregulated were *IDO1* (Indoleamine 2,3‐Dioxygenase 1)^31^; *LCP1* (Lymphocyte Cytosolic Protein 1)^32^; *BST2* (Bone Marrow Stromal Cell Antigen 2)^33^; and *CEACAM6* (Carcinoembryonic Antigen‐Related Cell Adhesion Molecule 6),^34^ which are all involved in cancer progression. Among the downregulated genes related to cancer progression were *CXADR* (Coxsackievirus and adenovirus receptor)^35^; *TACSTD2* (Tumor‐Associated Calcium Signal Transducer 2)^36^; *CTSF* (Cathepsin F)^37^; and, *ZNF43* (Zinc Finger Protein 43)^38^ (Figure 1D). Taken together these data support the notion that Siglec‐XII expression may facilitate late‐stage carcinoma progression in humans. A full list of differentially expressed genes is in Supplementary Data 1.

### 3.2 Enrichment analysis shows that similar gene sets are upregulated in Siglec‐XII expressing cell lines and Shp2‐expressing cell lines

To query which molecular pathways are altered by Siglec‐XII expression status, we performed gene set enrichment analysis (GSEA) on the expression profiles produced for each of the two PC3 cell lines. The GSEA result shows that transcriptional changes in Siglec‐XII expressing cells affect the expression of many gene sets found in the Molecular Signature Database (MSigDB).^22^ Of relevant interest are gene sets found in the Oncogenic Signatures collection, which were generated based on data produced by perturbing known cancer genes. The most dramatically enriched oncogenic signatures in Siglec‐XII expressing cells include a set of genes altered in KRAS‐addicted cancers,^39^ and a set of TAZ‐associated genes found to be enriched in high‐grade tumors.^40^

To predict whether these gene set enrichments may be related to Siglec‐XII activation of Shp2 signaling, we performed the same analysis on gene expression profiles of cancer cell lines that were found to be either dependent on Shp2 or independent of Shp2 in the development of resistance to MEK inhibition.^41^ Expression data for these samples was downloaded from the Gene Expression Omnibus (NCBI GEO accession number GSE121117). This analysis revealed that many of the same sets of genes that are enriched in Shp2‐dependent cancers, are also enriched in our PC‐3 cells with forced expression of Siglec‐XII (Figure 1D). The full results of the enrichment analysis are available in the supplementary data.

### 3.3 Enhanced expression and unexpectedly high frequency of Siglec‐XII in carcinomas

**Figure 2:**
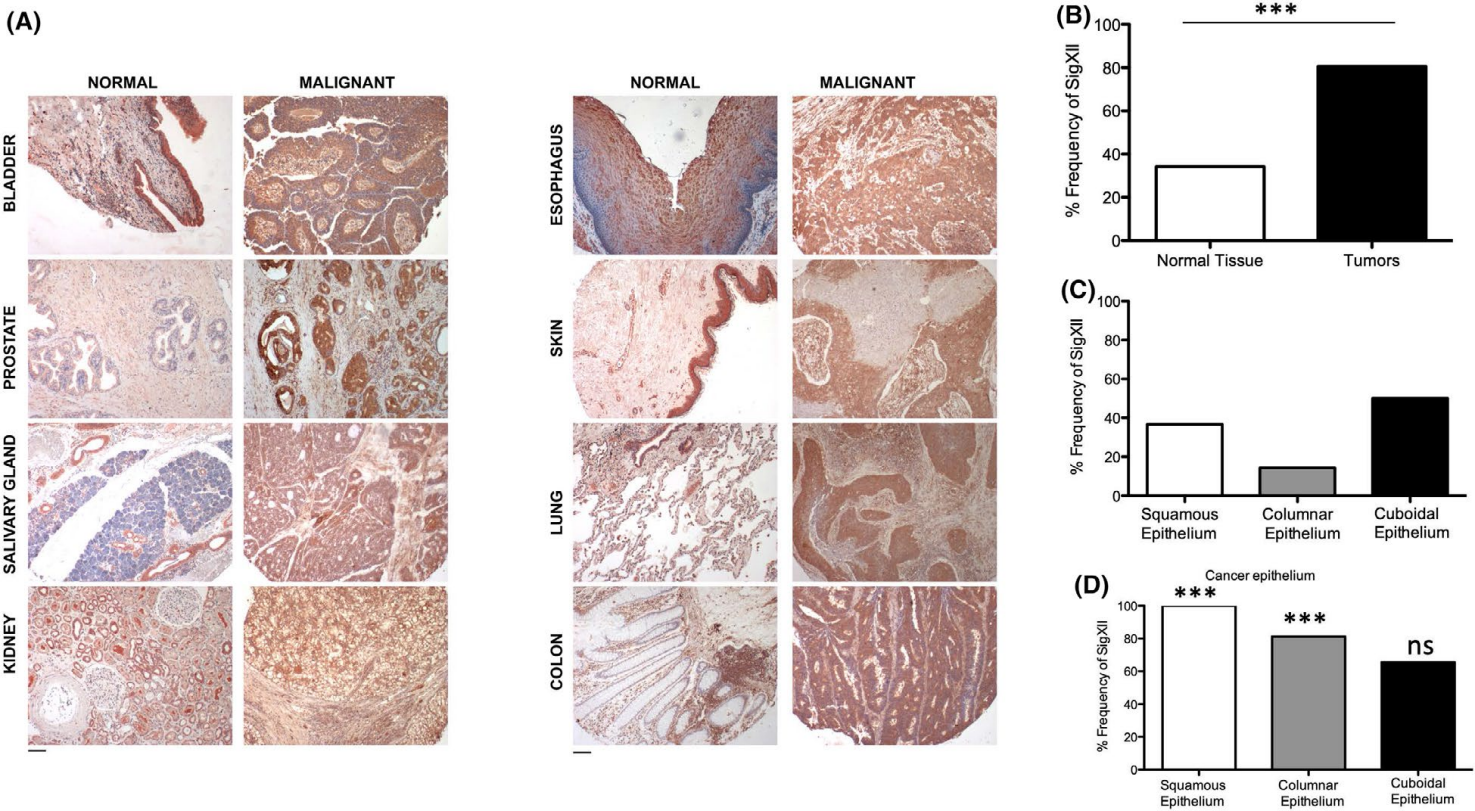
Enhanced Expression and Unexpectedly High Frequency of Siglec‐XII in carcinomas. A, Expression of Siglec‐XII studied in normal (benign) and cancer (malignant) human tissues using mouse monoclonal antibody clone 276 (See Materials and Methods). Representative examples of positive samples are shown. B, Frequency of Siglec‐XII detection on normal and cancer tissues (n = 97 for normal tissues and n = 85 for tumor samples, ***p value <0.001). C, Normal epithelium divided into squamous (n = 35), columnar (n = 14), and cuboidal (n = 34). D, Carcinoma epithelium also divided into squamous (n = 22), columnar (n = 16), and cuboidal (n = 32)

Immunohistochemical analyses for Siglec‐XII showed low to moderate level expression in normal epithelia in a commercially available normal multi‐tissue array with sample positivity of ~35%. As the majority of human genomes harbor a homozygous null‐state abrogating full‐length protein expression, this low frequency is as expected. In contrast, in a multi‐tissue array from the same source with multiple malignancies, we found an abundance of expression in carcinomas (malignancies arising from epithelia) (see examples in Figure 2A), with a much higher than expected frequency of Siglec‐XII in cancers (~80%) as compared to normal tissue (Figure 2B,C). Remarkably, 100% of the squamous cell carcinomas were positive (Figure 2D). This result was obtained from a mixed population group aged between 21 and 75 years. For this immunohistochemical analyses anti‐Siglec‐XII antibody clone 276 was used, which have been used and characterized earlier.^11^ For analysis of subsets of tumor types, the samples were divided into squamous, columnar, cuboidal, neural, and “uncategorized” (which included endothelium, mesothelium, and endocrine glands). Between 10 and 34 of each of these categories for both carcinoma and normal tissues were included in analysis. A paired *t*‐test was used to determine significance between frequency of Siglec‐XII+staining in normal or carcinoma samples (*p* < 0.01). Tumors included in the “malignant” multi‐tissue array are all likely to be advanced stage carcinomas. Given the prognosis of advanced carcinomas, our finding implies that minority of individuals who can express full‐length Siglec‐XII may be at the highest risk for dying with advanced carcinomas. A second panel of tissues, obtained from an independent source, and subjected to similar staining, confirmed this finding (Table 1).

**Table 1:** Second tumor microarray confirms unexpected high frequency of Siglec‐XII expression in carcinomas

	Total	Expressors	% Positive
Squamous Carcinomas			
Cervix	21	19	90%
Lung	18	17	94%
Skin	3	3	100%
Esophagus	3	1	33%
Head and Neck	17	15	88%
Salivary gland	4	3	75%
Adenocarcinomas			
Stomach	24	10	42%
Colon and Rectum	22	14	64%
Prostate	6	5	83%
Kidney	16	13	81%
Pancreas	5	2	40%
Breast	14	14	100%
Endometrium	16	15	94%
Other Malignancies			
Bladder	14	11	79%
Melanoma	9	8	89%
Hepatocellular carcinoma	13	4	31%
Lymphoma	16	4	25%
Bone sarcoma	17	8	47%
Esophagus	4	1	25%
Salivary gland	2	2	100%
Skin	3	1	33%
Bladder	4	1	25%
Kidney	5	1	20%
Lung	4	1	25%
Liver	3	0	0%
Breast	3	2	67%
Colon	4	0	0%
Pancreas	2	1	50%
Prostate	2	0	0%
Stomach	2	1	50%
Lymph node	3	0	0%

As shown in the main text, advanced carcinomas are more than twice as likely to express Siglec‐XII protein. To independently verify this unexpected result, a second panel of tissues obtained from another source was subjected to immunohistochemical analysis, including samples categorized as squamous carcinomas, adenocarcinomas, and other malignancies. As shown in this table the total frequency of Siglec‐XII expression in these carcinoma samples was 70% (166/238), while the frequency expression in normal control tissues included in the analysis was 27% (11/41), in alignment with the expected frequency of individuals in the global population with intact Siglec‐XII open reading frame. These data are categorized by cell type of origin. Notably with the exception of melanomas, other non‐epithelial malignancies such as lymphomas, sarcomas, and hepatocellular carcinomas had lower rates of expression, consistent with the population frequency of the frameshift allele.

### 3.4 No correlation between *SIGLEC12* genomic status and frequency or progression of early stage cancers

**Figure 3:**
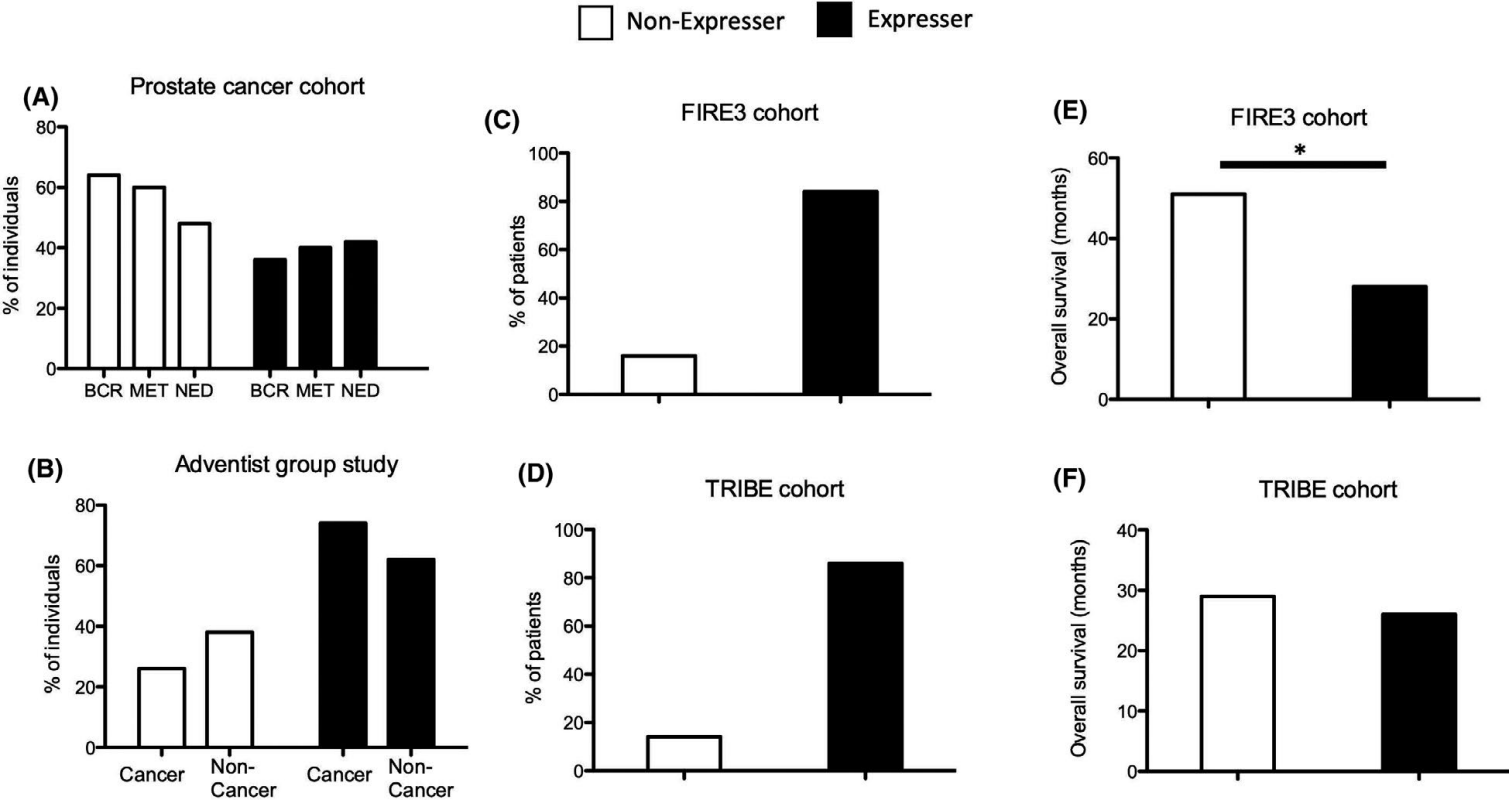
Correlation between *SIGLEC12* genomic status and frequency or progression, only of late‐stage cancers. A, Prostate cancer patients diagnosed with PSA test followed up after 5 years. (NED‐No evidence of disease: n = 84, BCR‐ Biochemical Cancer Recurrence: n = 28 and Met‐Metastasis: n = 10). B, Seventh‐Day Adventist population where environmental risk factors for cancer are minimal. The percentage of patients with cancer and without cancer is shown to be either *SIGLEC12*‐/‐ (non‐expresser, n for cancer = 14, n for non‐cancer = 20) or *SIGLEC12*+/‐ and *SIGLEC12*+/+ (expresser, n for cancer = 40 and n for non‐cancer = 33). C and D, Percentage of patients who are Siglec‐XII expressers (*SIGLEC12*+/‐ and *SIGLEC12*+/+) or non‐expressers (*SIGLEC12*‐/‐) in the FIRE3 and TRIBE stage IV colorectal cancer cohorts (FIRE3 cohort: expresser n = 85, non‐expresser n = 16 and TRIBE cohort: expresser n = 177, non‐expresser n = 27). E and F, Overall survival of colorectal cancer patients that are Siglec‐XII expressers versus non‐expressers (*p value <0.05)

Next, we asked if Siglec‐XII expression predicts early carcinoma risk or progression in a well‐defined population. We had already reported that the incidence of prostate cancer was not different between men with different *SIGLEC12* genotypes.^11^ From the same cohort, there is now a minimum of 5‐year follow‐up available for many of these patients categorized into no evidence of disease (NED); Biochemical recurrence (BCR) and Metastasis (Met). There was no clear correlation of *SIGLEC12* status with the progression of these early stage carcinomas (Figure 3A). Of course, most of these cases were originally diagnosed by a measuring prostate‐specific antigen (PSA), which picks up many early stage cases that never progress in the lifetime of the individual.^42^ Indeed, if we compare the patients with a poor outcome, versus those with no evidence of disease recurrence following prostatectomy (NED), we find that most of the patients (84 out of 122) detected by PSA screening did not have disease progression at the time of follow‐up.

Seventh‐day Adventists are members of a religious sect that do not smoke or consume alcohol and have a largely vegetarian diet with limited intake of red meat.^43^ As usual risk factors for cancer are limited, carcinoma incidence is much lower than in the general population. We genotyped the common *SIGLEC12* frameshift insertion mutation on genomic DNA from the peripheral blood cells of 54 Seventh‐day Adventist cancer patients and 53 non‐cancer patients (age and sex‐matched). While we found more Siglec‐XII expressers in the cancer group, this trend suggesting that Siglec‐XII expressers may be more prone to develop carcinomas was not statistically significant (Figure 3B). Notably, many of these cancers were diagnosed at an early stage. Taken together, the data above suggest that the genomic status of *SIGLEC12* may not be correlated with the early cancer risk, but rather with late progression.

### 3.5 High frequency of *SIGLEC12* expression in advanced colorectal cancer cohort and correlation with overall survival

Given the lack of significant correlation between *SIGLEC12* status and carcinoma risk or early stage carcinomas, we reasoned that there might instead be a correlation with late‐stage cancers. Indeed, in two stage IV colorectal cancer cohorts FIRE3 (592 patients from Germany and Austria)^44^ and TRIBE (508 patients from Italy)^45^ >80% of patients expressed Siglec‐XII based on the frameshift mutation (Figure 3C,D). This recapitulates our initial immunohistochemistry‐based findings. Furthermore, to see if *SIGLEC12* status has prognostic value, we checked overall survival in relation to Siglec‐XII expression. Interestingly, in FIRE3 the overall survival increased from 28 months to 51 months between Siglec‐XII expressers and non‐expressers (Figure 3E,F), that is, a correlation between Siglec‐XII expression and poor prognosis in late‐stage colorectal cancer.

### 3.6 Further evidence for selection at the *SIGLEC12* locus

**Figure 4:**
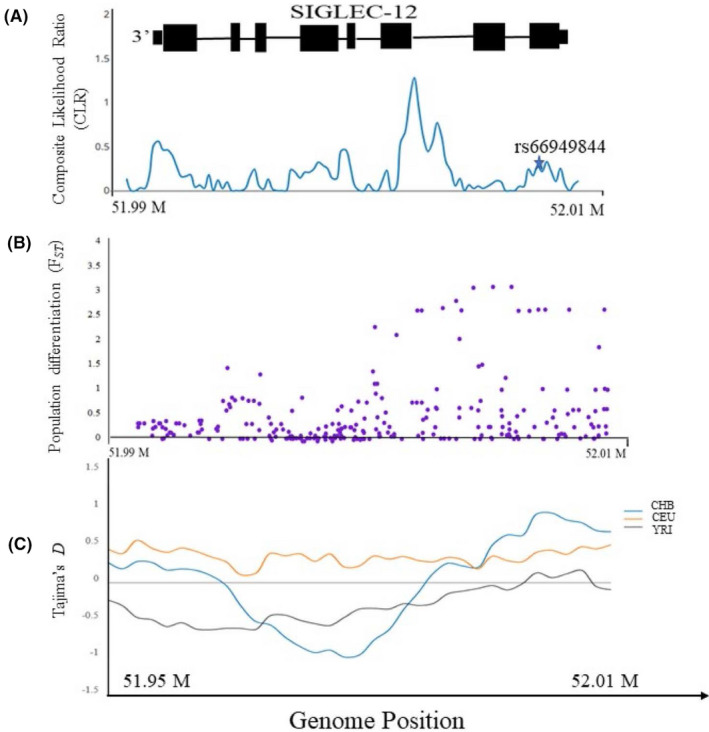
Population Genetic Analysis shows signatures of selection in and around the *SIGLEC12* locus. A, Signatures of “Selective sweep” in *SIGLEC12* in human population. The composite likelihood ratio (CLR) test of selective sweep based on the site‐frequency spectrum (SFS) is shown in blue. The star shown in the figure denotes the location of frameshift mutation. (Note. Schematic representation of *SIGLEC12* gene on top). B, Estimation of Population differentiation “F*_ST_*” (global) in three human populations (CHB_CEU_YRI). The purple dots represents F*_ST_* values. C, Estimation of Tajima's *D* in and around region of *SIGLEC12* in three human population are shown in different colors (Blue = CHB, Orange = CEU, and Grey = YRI)

Earlier work suggested that this locus might be undergoing selection favoring the null state.^17^ To test this hypothesis, we examined the population level genetic variation and evidence of natural selection in and around the *SIGLEC12* locus and carried out tests that aimed to detect a selective sweep,^29^ deviation from neutrality,^25^ and population differentiation^46^ in three ethnic groups (YRI, CHB, and CEU).^24^ Analysis of site‐frequency spectrum provided a composite likelihood ratio indicative of a soft “selective sweep” acting on the gene throughout the overall human population (Figure 4A). Additionally, the common frameshift mutation (rs66949844) was present adjacent to this area. We also estimated population differentiation (measured as Wright's index of fixation; F*_ST_*) and found moderately high F*_ST_* values (0.3) compared to the average F*_ST_* for genome‐wide autosomal markers throughout the human population.^47^ The high F*_ST_* value indicates stark differentiation of populations, which suggests directional selection (see Figure Legend, Figure 4B). Furthermore, we found an excess of rare alleles relative to a model of neutral evolution as indicated by negative Tajima's D values (Figure 4C); especially in CHB and YRI (African ancestry population). Individually none of these signals were very strong, but together, they suggest selection for the null state (note that ongoing selection for a null state would favor inactivating mutations, which would tend to mask the usual signatures of a selective sweep).

### 3.7 Dot blot analysis of bladder epithelial cells for detection of Siglec‐XII status

**Figure 5:**
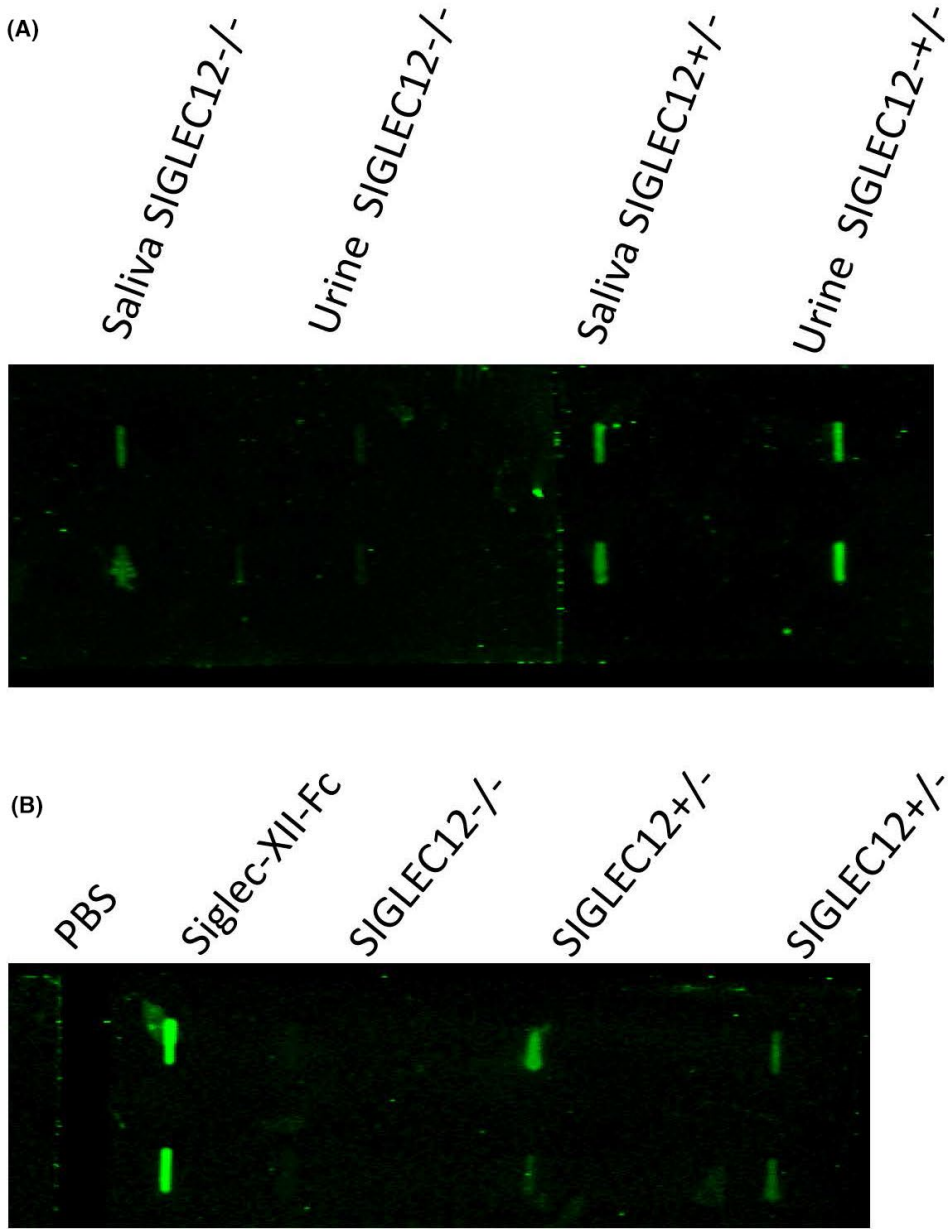
Simple analysis of urine allows screening for Siglec‐XII protein expression status. A, Urine and saliva samples were obtained from healthy individuals and used for checking protein expression of Siglec‐XII by the dot blot. B, Urine samples from multiple healthy donors were used to check protein expression of Siglec‐XII. One typical example is shown. (The whole blot was corrected uniformly for brightness using Photoshop, to match the visual appearance)

All the population studies above were handicapped by the fact that in addition to the common frameshift insertion mutation, we found other less common mutations that would nullify Siglec‐XII expression. For example, another deleterious mutation (rs16982743) was observed at a global frequency of 18.6% that changes glutamine to a stop codon (Q>*) at the 29th position.^12^ Thus, biallelic whole exome sequencing of *SIGLEC12* genomic DNA would be required for rigorous population studies. Even this approach could be confounded by selection for noncoding mutations that suppress gene expression in a given allele with an intact open reading frame. In addition, there is evidence for an alternately spliced truncated form of the protein.^16^ To facilitate future population and cancer cohort studies, it would be useful to have a simple assay to rapidly detect all mutations abrogating expression, without the need to do whole exome sequencing. We took advantage of the fact that among normal epithelial tissues tested by IHC, Siglec‐XII was expressed in bladder epithelium, kidney tubules and salivary gland ducts, and detected the expression of Siglec‐XII in cells isolated from saliva and urine (Figure 5A). It was determined via buccal swab genomic analysis that the *SIGLEC12* genomic status (*SIGLEC12* +/‐ or ‐/‐) correlates with either Siglec‐XII expression (+/‐) or no expression (‐/‐). As expected, Siglec‐XII expression in cells obtained from the urine of multiple healthy donors showed expression of Siglec‐XII in the +/‐ genotypes and no expression in the Siglec‐XII null genotypes. While there was a significant background in samples from saliva, results from dot blot screening of urinary cells were very clean (a typical example is shown in Figure 5B).

## 4 DISCUSSION

We focused our initial work on a common polymorphic frameshift mutation in human populations with an allele frequency ranging from 38% in sub‐Saharan Africans to 86% in the Native American population.^11^ An earlier study^17^ suggested selection on *SIGLEC12* based on the inactivating mutation rs16982743. However, another frameshift mutation (rs66949844) was present in the human population at an allele frequency of 59%.^48^ Overall, this region of *SIGLEC12* showed reduced genetic diversity, which was supported by a sweep scan.^29^ These findings were concordant with results from a study in six different human populations^49^ showing a soft sweep in a region of *SIGLEC12*. The presence of excess rare alleles in and around a genomic region is also an indicator of a low level of population differentiation^47^, ^50^ further indicating the presence of purifying selection or balancing selection. Negative selection in S*IGLEC12* region was also evident from the result of Tajima's D (TD) especially in the YRI population (African ancestry).

**Figure 6:**
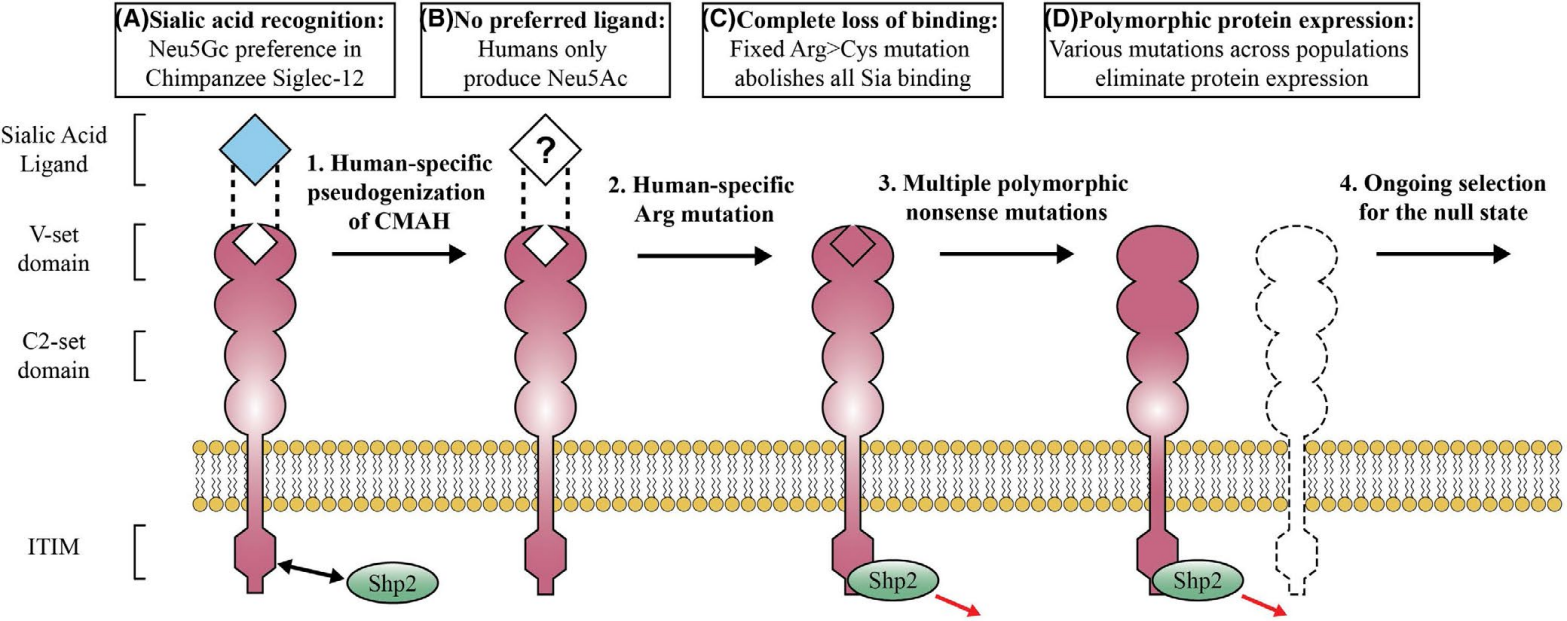
Proposed evolutionary history of human Siglec‐XII. A, The last common human‐chimpanzee ancestor and modern chimpanzees had a functional CMAH enzyme and an abundance of Neu5Gc‐terminated cell‐surface glycans. Chimpanzee Siglec‐12 recognizes Neu5Gc through an arginine‐dependent binding pocket in its terminal V‐set domain. B, After divergence from chimpanzees CMAH was completely inactivated in human ancestor, leaving Siglec‐12 with no endogenous ligand. C, Another unknown evolutionary event fixed a mutation in the critical arginine rendering human “Siglec‐XII” incapable of binding any sialic acids, however, the full‐length protein continues to recruit Shp2 and alter gene expression. D, Modern humans are experiencing purifying selection acting to increase the frequency of common null‐state alleles across population

Previous studies showed that while the non‐Sia‐binding Siglec‐XII can be expressed in *SIGLEC12* mutated PC‐3 human prostate cancer cells, efforts to transfect the chimpanzee version of *SIGLEC12* or the arginine‐restored version of human *SIGLEC12* were not successful.^11^ This could be either due to rapid turnover or selection against expression in vitro. Regardless, the non‐Sia‐binding full‐length human Siglec‐XII is clearly different functionally, allowing persistent surface expression in malignant cells by as yet unknown mechanisms. Chimpanzee and arginine‐restored human *SIGLEC12* both display a preference for binding *N*‐Glycolylneuraminic acid (Neu5Gc) (Figure 6A)^8^ which is absent in humans due to a homozygous fixed deletion in the gene *CMAH*.^51^ After losing its preferred ligand in an ancestral prehuman species, it is possible that Siglec‐12 was susceptible to exploitation by a Neu5Gc‐presenting pathogen (Figure 6B) or some other harmful form of activation, driving the fixation of the arginine mutation to produce the non‐Sia‐binding full‐length Siglec‐XII found in humans today (Figure 6C). It is worth pointing out that the reason the arginine mutation was fixed in humans remains unknown, and the consequences of the Sia‐binding chimpanzee Siglec‐12 deserve further study to contribute to our understanding of this evolutionary scenario. Finally, with the unusual derived trait of post‐reproductive life span in modern humans, we propose that selection for survival in late life is driving the complete loss of the human *SIGLEC12* gene, as evidenced by the genomic signatures we report. Ongoing selection for null‐state alleles may be acting to relieve the increased risk of advanced carcinomas produced by the archaic nonfunctional Siglec protein (Figure 6D).

It is also important to reemphasize that the arginine and frameshift mutations of Siglec‐XII do not occur in chimpanzees. Humans and chimpanzees are very similar in terms of genomic sequences but different phenotypically. Remarkably, while cancers are common in humans, few are reported in chimpanzees, and are usually lymphomas or soft tissue tumors, unlike those that arise in humans, who are instead prone to epithelial carcinomas.^1^, ^2^, ^3^ Here, immunohistochemistry analyses indicate that Siglec‐XII is highly expressed in advanced carcinomas, as compared to normal epithelium. Considering the multiple mutations reported^11^, ^12^ and others possible in the population, the overall expression in ~35% in normal samples seems reasonable to represent the general mixed population. On the contrary, the high abundance of Siglec‐XII in advanced carcinomas is remarkable, as is the high frequency of expression at >80%, in epithelial carcinomas. A second panel shows that certain types of carcinomas are even more likely to be found in Siglec‐XII expresses; all squamous carcinomas show an 88% frequency, with lung showing 94% and cervix 90% (see Table 1).

To explore molecular mechanisms of Siglec‐XII in cancer progression, we compared RNA expression patterns between *SIGLEC12* null PC‐3 prostate cancer cells with and without transfection with a construct encoding Siglec‐XII. One of the top hits among the upregulated genes in RNA‐Seq was IDO‐1 (Indoleamine 2,3‐dioxygenase 1), an enzyme involved in conversion of tryptophan to kynurenine metabolites. This enzyme is highly upregulated in many types of cancers. It is known that a decrease in the levels of tryptophan and an increase in the levels of kynurenine leads to immunosuppression and enhanced tumor growth.^31^, ^52^, ^53^ The molecular mechanisms for the effects of IDO‐1 overexpression point toward maintenance of immunosuppression in tumor microenvironment, due to depletion of effector T cells and enrichment of regulatory T cells.^31^ There has been a recent focus on IDO‐1 targeting through small molecule inhibitors in preclinical and clinical settings.^52^, ^54^

While Siglec‐XII has lost its Sia‐binding property, it still has the ability to recruit Shp1 and Shp2.^9^ Shp2 (encoded by *PTPN11*) is a well‐characterized oncogene that elicits cell growth, proliferation, tumorigenesis, and metastasis.^55^ Over‐activation and activating mutations of Shp2 are known to be involved in breast cancer, leukemia, and gliomas.^55^, ^56^, ^57^

While not the primary objective of this study, we used RNA‐sequencing data from our Siglec‐XII expressing PC‐3 cells to briefly investigate the hypothesis that Siglec‐XII expression enhancers tumor growth via Shp2. Using Gene Set Enrichment Analysis, we identified which pathways are altered by Siglec‐XII protein expression. This analysis revealed that among the most dramatically enriched pathways are KRAS and YAP/TAZ. Notably, when we conducted a parallel analysis from a previously published transcriptomic study of Shp2‐dependence the same pathways were the most highly enriched.^41^ Upregulation of these well‐known oncogenic pathways in individuals with an intact *SIGLEC12* allele may explain the molecular mechanisms underpinning our discovery of increased frequency of Siglec‐XII protein in advanced carcinomas.

We also performed population studies on four cancer cohorts. The first was a prostate cancer cohort we had studied earlier.^11^ While a 5‐year follow‐up for 122 patients was recorded in this cohort, we still did not see any positive correlation between *SIGLEC12* pseudogenization and outcome. One reason for this negative result may be that out of 122 patients only 10 developed metastasis (poor prognosis) and this might not be a sufficient number to find the relevance of *SIGLEC12* in prognosis. The second cohort we tested was a Seventh‐day Adventist group and the lack of correlation could be due to two reasons. First, most of the cancer patients in this group represented early stage cancer, where the effect of Siglec‐XII is not pronounced. Second, many cancer risk factors such as intake of red meat, smoking, drinking alcohol etc., are minimal in this cohort, so it might be that Siglec‐XII plays a role only when other obvious risk factors are involved. Overall, it appears that Siglec‐XII does not play a role in early stage carcinomas. In other populations, we discovered that the null state of the gene affects the prognosis of advanced carcinomas. Therefore, Siglec‐XII expression is more likely to contribute to the advancement of benign neoplasia to deadly malignancies.

According to the well‐established theoretical concept, natural selection occurs in prereproductive or reproductive individuals.^58^ However, humans are a rare species that have prolonged post‐reproductive life span (PRLS), and according to the “grandmother hypothesis” inclusive fitness of infertile elderly caregivers can determine the fate of helpless grandchildren.^59^, ^60^ We report selection acting on the *SIGLEC12* locus in human populations. This could be caused by deleterious fitness consequences of advanced carcinomas, which mostly occur late in middle to late life. To the best of our knowledge, our work is the first potential example of inclusive fitness effects selecting for cancer suppression, supporting a function for PRLS in humans. In contrast, an expansion in the number of p53 genes maybe providing late life protection against cancer risk in long‐lived elephants.^61^ However, elephants do not have a PRLS, so the underlying selection mechanism must be different.

This first study of a very unusual phenomenon raises even more questions than answers. We do not know if there is still any definite ligand for Siglec‐XII. It does not bind with Sias, but we cannot rule out its interaction with another unknown ligand(s). Conversely, we can also consider the hypothesis that this is a constitutively active receptor, which does not need any ligand for its activation. This aspect of Siglec biology is not extensively studied. Second, we did not study the signaling pathways mediated by SiglecXII‐Shp2 axis. Third, we have not yet done the gene expression analysis in PC‐3 cells with Shp2 inhibitors. Moreover, a knockdown of *SIGLEC12* in a Siglec‐XII expressing cell line will be useful. These are important aspects of Siglec‐XII biology, which will be focused in further studies. Regardless, we have previously noted that triggering of endocytosis by antibodies against this receptor can deliver toxins into the cell.^11^ In analogy to the targeting of Siglec‐3/CD33 human leukemias,^62^ a similar approach could be taken for treatment of late‐stage carcinomas. Our simple urine screen should be of value in these and other clinical studies.

## CONFLICT OF INTEREST

The authors declare no competing interest with the content of this manuscript.

## AUTHORS CONTRIBUTION

SSS, NV, and AV conceived the idea and designed the experiments. SSS, RD, MV, NK, ALV, WZ, HJL, TLJ, RJL, GF, NV, GSF, and CW performed the experiments and analyzed the results. NV and AV supervised the work. SSS, MV, NK, and AV wrote the manuscript. All authors read the manuscript and approved it.

## Supporting information

Dataset S1
